# Correction of the NSE concentration in hemolyzed serum samples improves its diagnostic accuracy in small-cell lung cancer

**DOI:** 10.18632/oncotarget.27664

**Published:** 2020-07-07

**Authors:** Sylvia A.A.M. Genet, Esther Visser, Ben E.E.M. van den Borne, Maggy Youssef-El Soud, Huub N.A. Belderbos, Gerben Stege, Marleen E.A. de Saegher, Federica Eduati, Maarten A.C. Broeren, Joost van Dongen, Luc Brunsveld, Daan van de Kerkhof, Volkher Scharnhorst

**Affiliations:** ^1^Laboratory of Chemical Biology, Department of Biomedical Engineering and Institute for Complex Molecular Systems, Eindhoven University of Technology, Eindhoven, The Netherlands; ^2^Catharina Hospital Eindhoven, Eindhoven, The Netherlands; ^3^Máxima Medical Center, Eindhoven/Veldhoven, The Netherlands; ^4^Expert Center Clinical Chemistry Eindhoven, Eindhoven, The Netherlands; ^5^Amphia Hospital, Breda, The Netherlands; ^6^Sint Anna Hospital, Geldrop, The Netherlands; ^7^Sint Jans Gasthuis, Weert, The Netherlands

**Keywords:** small-cell lung cancer, protein tumor markers, neuron-specific enolase, hemolysis correction equation

## Abstract

Neuron-specific enolase (NSE) is a well-known biomarker for the diagnosis, prognosis and treatment monitoring of small-cell lung cancer (SCLC). Nevertheless, its clinical applicability is limited since serum NSE levels are influenced by hemolysis, leading to falsely elevated results. Therefore, this study aimed to develop a hemolysis correction equation and evaluate its role in SCLC diagnostics.

Two serum pools were spiked with increasing amounts of hemolysate obtained from multiple individuals. A hemolysis correction equation was obtained by analyzing the relationship between the measured NSE concentration and the degree of hemolysis. The equation was validated using intentionally hemolyzed serum samples, which showed that the correction was accurate for samples with an H-index up to 30 μmol/L. Correction of the measured NSE concentration in patients suspected of lung cancer caused an increase in AUC and a significantly lower cut-off value for SCLC detection when compared to uncorrected results.

Therefore, a hemolysis correction equation should be used to correct falsely elevated NSE concentrations. Results of samples with an H-index above 30 μmol/L should not be reported to clinicians. Application of the equation illustrates the importance of hemolysis correction in SCLC diagnostics and questions the correctness of the currently used diagnostic cut-off value.

## INTRODUCTION

Neuron-specific enolase (NSE) is a dimeric metalloenzyme which functions as a cell specific isoenzyme of the glycolytic enzyme enolase [1]. It is comprised of γγ homodimers and αγ heterodimers that are predominantly expressed in mature neurons and cells of neuronal origin, although the αγ heterodimer can also be found in erythrocytes and platelets and released upon hemolysis [2, 3]. Previously, the NSE concentration in serum was shown to correlate with tumor burden, metastasis and treatment response in tumors of neuroendocrine origin such as small-cell lung cancer (SCLC) [1, 4, 5]. Furthermore, improved discrimination of the two main lung cancer subtypes, SCLC and non-small cell lung cancer (NSCLC), was achieved when applying a diagnostic cut-off value of 25 ng/mL NSE or analyzing multiple protein tumor markers such as NSE and progastrin-releasing peptide (ProGRP) at the same time [4, 6–10].

Considering the use of NSE in lung cancer diagnostics and the medical actions that may follow, accurate and reliable quantification of NSE is of main importance. Nevertheless, interpretation of the results can be challenging since the presence of the NSE αγ heterodimer in erythrocytes and platelets can induce falsely elevated outcomes if hemolyzed samples are measured [2, 4, 11–14]. Without correcting for the influence of hemolysis on the measured NSE concentration, samples exceeding a hemolysis threshold should either be rejected, recollected or commented regarding reliability [2]. However, previous studies evaluating the prognostic value of NSE in lung cancer diagnostics did not apply exclusion criteria [15–18] or did not include the effect of hemolysis on the measured NSE concentration as such, while other factors that could influence serum tumor marker concentrations were addressed [5, 6, 10, 19]. One study did reject hemolytic samples from further analysis, although neither the hemolysis detection method nor the acceptable degree of hemolysis were described [20].

Previously, various correction equations for NSE detection in neonatal serum samples suffering from brain injuries were established [3, 11]. However, since the NSE concentration in erythrocytes of newborns substantially differs from that of adults (± 25 μg NSE/mg hemoglobin (Hb) and ± 25 ng NSE/mg Hb respectively), these equations cannot be used in lung cancer diagnostics [2, 3]. Other earlier developed correction equations for NSE quantification in adults were based on detection methods prone to assay interference [2]. Therefore, this study aimed to develop, validate and apply a hemolysis correction equation that nullifies the effect of hemolysis on NSE quantification in samples of adult patients. Using this equation, the effect of hemolysis correction on the NSE cut-off value in SCLC diagnostics was evaluated and the maximum acceptable degree of hemolysis for reliable correction was established.

## RESULTS

### Derivation of a hemolysis correction equation

Two serum pools with initial NSE concentrations of 33.3 ng/mL (pool 1) and 18.1 ng/mL (pool 2) were spiked with hemolysate obtained from five different individuals (pool a–e) [1, 6]. The degree of hemolysis, represented by the hemolysis index (H-index) [μmol/L], and the measured NSE concentration [ng/mL] showed a hemolysis-dependent increment in measured NSE concentrations in all pools (Figure 1A and Supplementary Table 1). The results of the individual pools were combined by transforming the data through the origin (0,0) with the use of ΔNSE (NSE_measured_ – intercept) and plotted as a function of the H-index (Figure 1B). With the use of linear regression, a line described by Equation 1 was derived.

**Figure 1:**
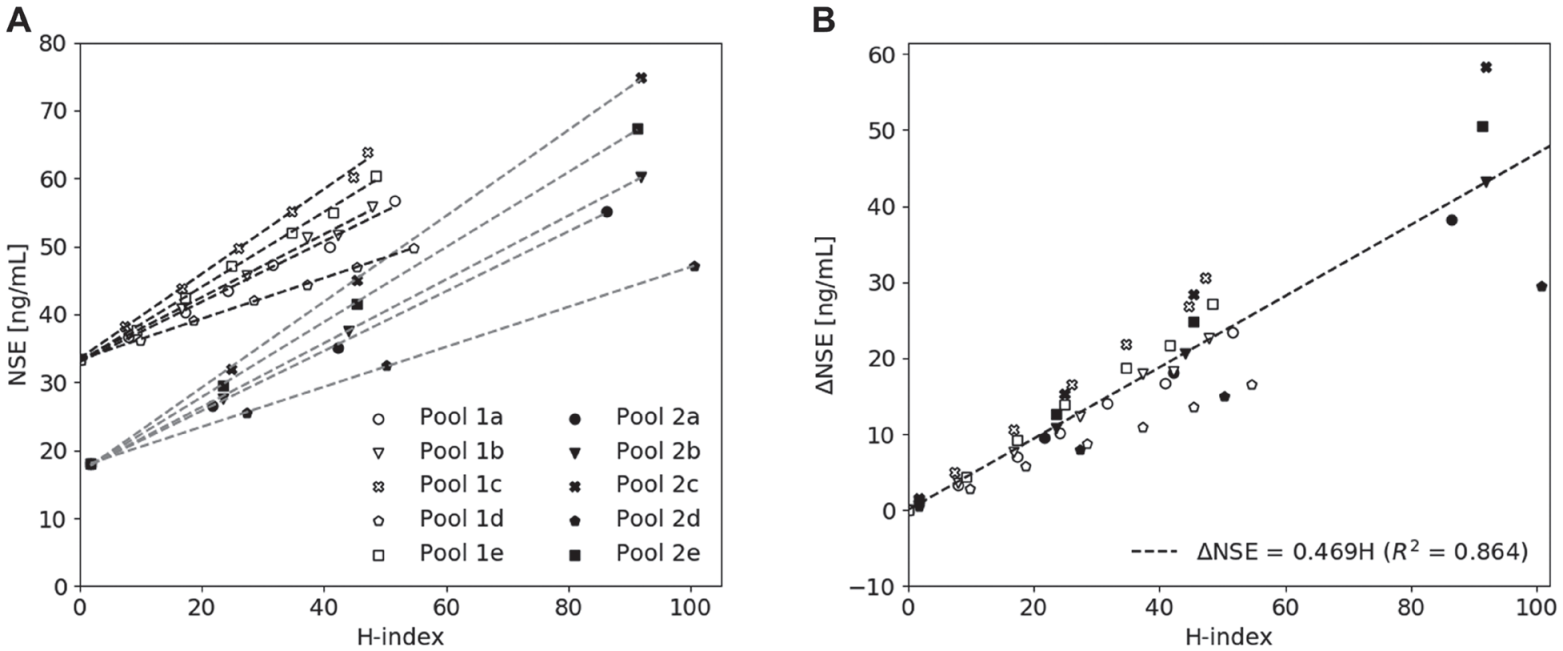
The influence of hemolysis on the measured NSE concentration. (**A**) A hemolysate spiking study showing a hemolysis-dependent elevation of measured NSE concentration in two serum pools spiked with increasing amounts of hemolysate derived from five individuals (a–e). (**B**) Derivation of the hemolysis correction equation by combining all pools and plotting the corresponding ΔNSE concentration as a function of H-index.

$$\left(1)\;\Delta \text{NSE}=0.469\;\text{H-index}({\text{R}}^{2}=0.864\right)$$

The measured NSE concentration, the slope of Equation 1 and the measured H-index were then combined to determine the corrected NSE concentration [ng/mL], as described by Equation 2.

$$(2)\;{\text{NSE}}_{\text{Corrected}}={\text{NSE}}_{\text{measured}}-0.469\;{\text{H-index}}_{\text{measured}}$$

### Validation of the hemolysis correction equation

The performance of the hemolysis correction equation (Equation 2) was evaluated by applying the correction on a training dataset (serum pool 1 and 2 spiked with hemolysate pool a–e) and a validation dataset (serum pool 1 and 2 spiked with hemolysate pool f-j). The difference between the corrected and baseline NSE concentration, the initial NSE concentration at H-index = 0 μmol/L, was plotted as a function of the H-index and showed an increase in standard deviation (SD) upon an increase in H-index (Figure 2A). The maximum tolerable error after correction was determined by using the total allowable analytical error (TEa). Here, values corrected within 2TEa were assumed to be clinically acceptable, therewith allowing a maximum difference between corrected and baseline values of ± 7.38 ng/mL when using a diagnostic cut-off value of 25 ng/mL [6, 21, 22]. As shown in Figure 2A, 95% of all samples with an H-index of 30 μmol/L or less could be corrected within 2TEa. Statistical testing confirmed that uncorrected NSE values were significantly different from baseline and corrected values (*p* < 0.0001), while no significant difference between baseline and corrected values could be seen (*p* = 0.72) (Figure 2B). Based on QQ-plot analysis (Figure 2C) and the Shapiro-Wilk test, differences between the baseline, uncorrected and corrected NSE values were not assumed to be normally distributed (*p* < 0.0001). When the corrected samples were divided into smaller subgroups created by categorizing the samples per 10 units of the H-index, the differences between the baseline values and the subgroups was still not significant (*p* ≤ 0.53, data was assumed to be normally distributed).

**Figure 2:**
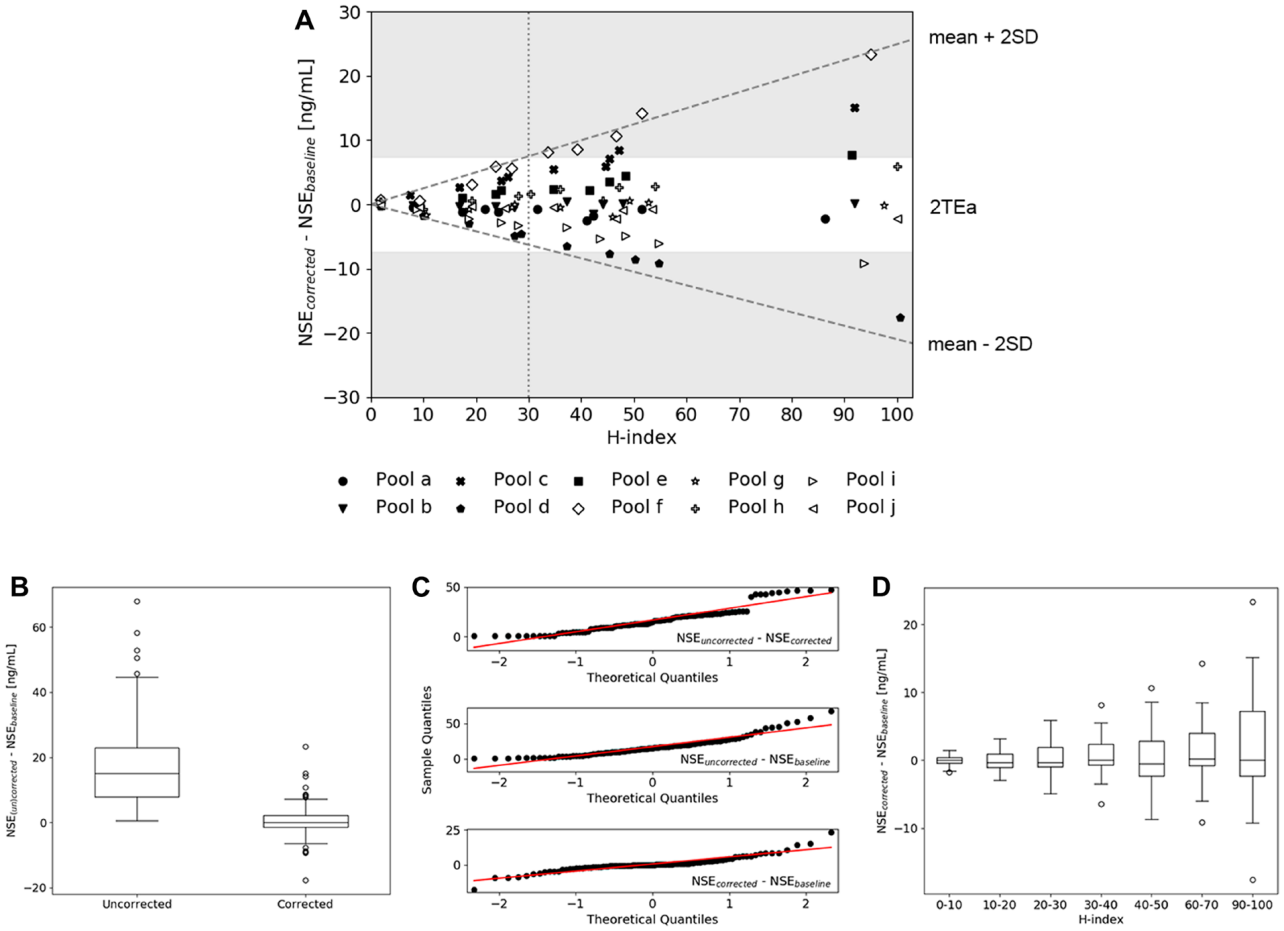
Validation of the hemolysis correction equation. (**A**) The difference between corrected and baseline NSE values of all hemolysate pools (a-j) spiked in serum pool 1 and 2 plotted as a function of H-index. The dashed lines indicate the mean ± 2SD, the dotted line illustrates the H-index level at which the mean ± 2SD equalizes 2TEa (H-index = 30). (**B**) Boxplots of the differences between (un) corrected and baseline NSE values, showing a significant difference between uncorrected and corrected values. (**C**) QQ-plots of the difference between baseline, uncorrected and corrected samples indicating non-normally distributed data. (**D**) Distribution of the differences between corrected and baseline NSE values, where the corrected group was divided into subgroups based on the H-index.

As shown in Supplementary Figure 1A, the results of both analytical platforms used in this study were comparable and within the analytical variation (CV_a_) of 3.8%. Furthermore, it was shown that the intra-individual variability of the slope, determined at three different collection time points (day 1 = time point 0, day 2 = 3 weeks after day 1, day 3 = 4.5 months after day 1), was within range of the CV_a_ (Supplementary Figure 1B).

### Applying the hemolysis correction equation in SCLC diagnostics

#### Characterization of participants

To evaluate the performance of the hemolysis correction equation on the detection of SCLC, the correction equation (Equation 2) was used within a relevant patient cohort (*n* = 316) consisting of a benign, SCLC and NSCLC group (Supplementary Figure 2 and Supplementary Table 2). Lung cancer was diagnosed in 241 patients of which 26 patients were having SCLC and 215 NSCLC. Lung cancer was excluded in 75 patients of which the alternative diagnoses are shown in Supplementary Table 2. The measured NSE concentration within the SCLC group was significantly different from the one of the benign and NSCLC group (*p* < 0.0001). After applying the hemolysis correction equation, the NSE concentration of all groups significantly decreased (*p* < 0.0001). Since the correction equation was shown to be applicable up to an H-index op 30 μmol/L, two patients could not be corrected appropriately and were therefore excluded from the analysis (Supplementary Table 2).

### ROC curve analysis before and after correction

To evaluate the influence of the correction equation on the ability to detect SCLC in a population consisting of a SCLC, NSCLC and benign group, ROC curves with the uncorrected and corrected NSE concentration were constructed *via* stratified bootstrapping (*n* = 100). This sampling method was used to make the results less dependent on outliers and allowed for proper quantification of the uncertainty of estimated statistics [23]. As shown in Figure 3A, correction of the NSE values induced a significant increase in AUC value, respectively 0.88 (0.81–0.96) before and 0.90 (0.83–0.97) after correction. Based on a QQ-plot analysis (Figure 3B) and the Shapiro-Wilk test, a normal distribution of the differences between the uncorrected and corrected AUC values was assumed (*p* = 0.11).

**Figure 3:**
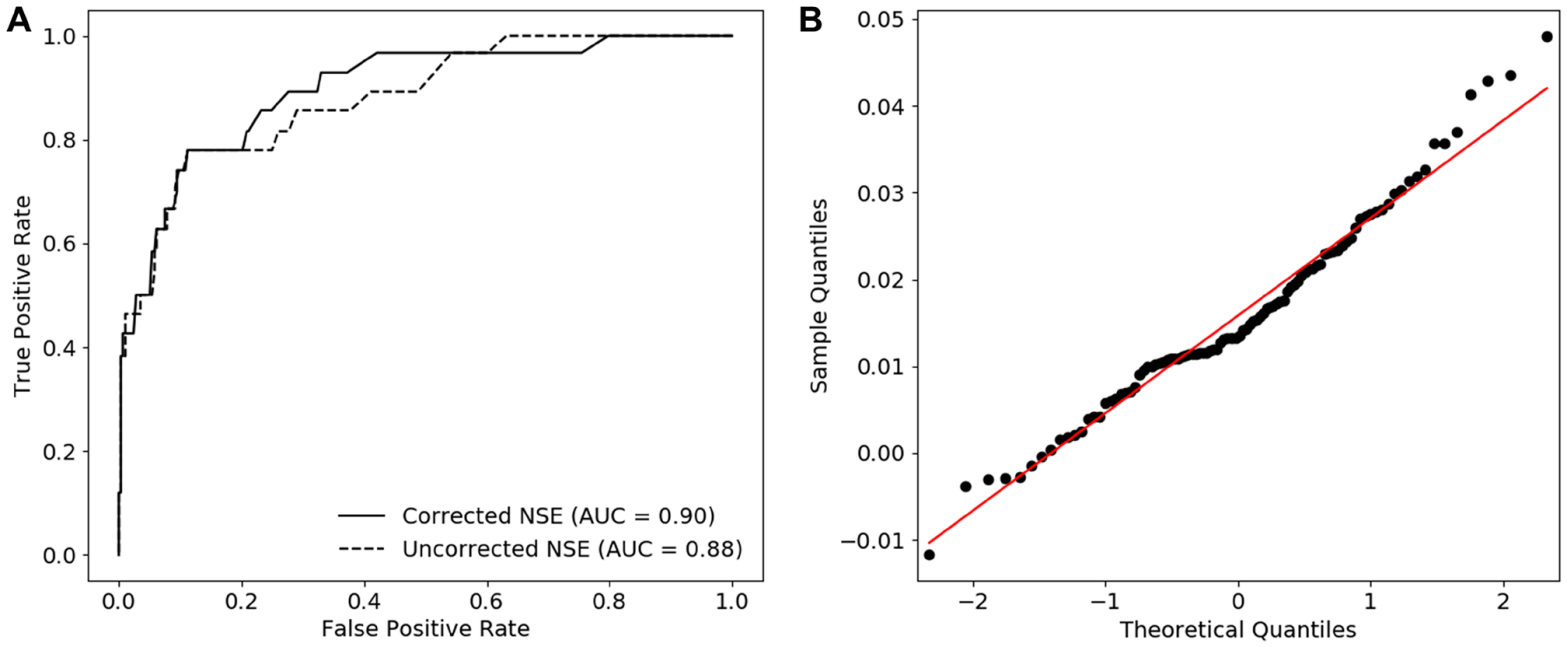
The diagnostic performance of corrected NSE in case of SCLC. (**A**) ROC curves constructed with the us of uncorrected and corrected NSE values and corresponding AUCs (median). (**B**) QQ-plots of the difference between the AUC of uncorrected and corrected NSE indicating non-normally distributed data.

### Assessment of the clinically relevant NSE cut-off value

Since the use of the hemolysis correction equation led to an increased AUC value, it is conceivable that the use of corrected NSE concentrations also requires a cut-off value different from 25 ng/mL, which is the current optimal threshold for SCLC diagnostics [6, 21]. For this reason, cut-off values were determined using the Youden’s J statistic in which the performance of a cut-off value is calculated assuming that sensitivity and specificity are diagnostically equally important. As shown in Figure 4A, evaluation of uncorrected NSE revealed an optimal cut-off value of 24.5 (24.5-26.4) ng/mL, which is in line with the previously published cut-off value of 25 ng/mL. However, assessment of the corrected NSE concentrations resulted in a significantly lower optimal cut-off value of 22.7 (16.0–22.8) ng/mL (*p* < 0.0001) (Figure 4B and 4C). Similar sensitivities (76.9%) and specificities (88.5% and 88.9% respectively) were achieved for the 24.5 ng/mL cut-off value without correction and the 22.7 ng/mL cut-off value with correction (Supplementary Table 3).

**Figure 4:**
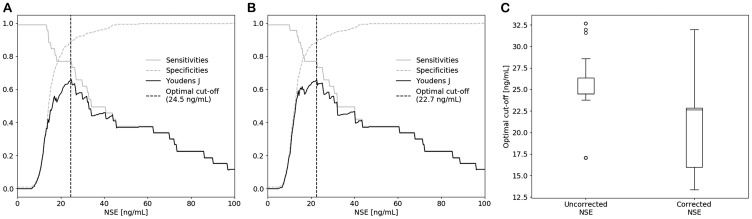
Optimal cut-off values for SCLC diagnostics of (**A**) uncorrected and (**B**) corrected NSE determined by the Youden’s J statistic and stratified bootstrapping. (**C**) Boxplots of the optimal cut-off values, showing a significant decrease in cut-off value after correction.

## DISCUSSION

This study demonstrates the added diagnostic value of a hemolysis correction equation in the quantification of serum NSE in patients suspected of lung carcinoma. The correction equation was developed by using two serum pools with initial NSE concentrations above and below the currently used diagnostic cut-off value (25 ng/mL) and five hemolysate pools derived from healthy donors. As shown in Figure 1 and Supplementary Table 1, comparable slopes were obtained when one of the hemolysate pools (a–e) was spiked in either serum pool 1 or 2, indicating that the slope was independent of the initial NSE concentration of the serum pool. However, spiking of the different hemolysate pools (a–e) in both serum pool 1 and 2 did result in different slopes ranging from 0.294 to 0.632, indicating a more than two-fold inter-individual variability in erythrocytic NSE concentration. Even though this indicates that the correction equation induces a certain amount of error for patients that deviate from the average slope, the equation can still be a valuable additive to the current method of serum NSE quantification. Not only because the development of an individualized correction equation is too labor-intensive in clinical practice and would require the collection of both a serum and whole blood sample, but also because the correction equation derived in this study induced significantly improved accuracy. Application of the equation led to NSE concentrations that were identical to the baseline (*p* = 0.71), while the uncorrected values significantly differed from both the corrected and baseline values (*p* < 0.0001) (Figure 2B and 2C). As shown in Figure 2D, no significant difference between the mean of the baseline and the subgroups of the corrected samples was observed. However, since the inter-quartile range (IQR) and SD increased upon an increase in H-index (Figure 2A and 2D), the TEa was used to define an acceptable range in which the correction equation can be used. Within 2TEa, values are assumed to be clinically comparable. Therefore, samples within 2SD from the mean are corrected with 95% certainty, meaning that the correction equation can be applied to samples with an H-index between 0 and 30 μmol/L. With the use of this H-index range, 99.4% of all 316 samples in this study could be corrected accordingly and so the equation is expected to be applicable for nearly all samples measured in the clinic. If a sample exceeds the upper range, the results should not be reported and a new serum sample should be requested.

Application of the hemolysis correction equation in patients suspected of lung cancer led to an improved performance in separating the SCLC group from the benign and NSCLC group, as shown by the increase in AUC after correction (Figure 3). Evaluation of the optimal diagnostic cut-off value also showed the need to carefully reassess the cut-off value of 25 ng/mL in follow-up studies, since a significant decrease in optimal cut-off value (24.5 to 22.7 ng/mL) was observed after applying the correction equation (Figure 4). As shown in Figure 2C, the IQR of the optimal cut-off value substantially increased after correction, which indicates that a larger number of SCLC patients is required to draw hard conclusions on the optimal cut-off value in SCLC diagnostics.

As previously mentioned, very divers hemolysis correction equations are described in current literature and the equation developed in this study is again different [2, 3, 11]. This can be explained by multiple factors, such as the difference in hemolysis interference between newborns and adults or the variability between measurement systems used to detect hemolysis and serum NSE. In this study, NSE was quantified using two commercially available and widely used Cobas platforms with high precision and limited interference compared to other measurement systems used in previous studies [2, 24–26]. As shown in Supplementary Figure 1A, results on both Cobas platforms were identical and so the approach described here is transferable to other immunochemical and chemical platforms. For every analyzer or test kit combination, the correction equation should be verified.

In conclusion, this study demonstrates that a hemolysis correction equation improves diagnostic accuracy of serum NSE concentrations in patients suspected of lung cancer. A hemolysis correction equation is therefore suggested to be incorporated in NSE-based clinical decision making, bearing in mind that results of samples with an H-index above 30 μmol/L should not be reported to clinicians.

## MATERIALS AND METHODS

### Sample handling and storage

Whole blood of patients suspected of lung carcinoma was collected in an 8.5 mL BD Vacutainer SSTII Advance Plus Blood Collection Tube during routine venipuncture. The tubes were processed within one hour after collection by centrifugation for 10 minutes at 2683 g at 20°C. Serum was aliquoted in 2 mL VWR microtubes and stored at –80°C until further analysis.

Pure hemolysate pools were prepared by collecting heparinized whole blood samples of 10 healthy volunteers. Blood of one volunteer (pool f) was collected at three time points, day 1 = time point 0, day 2 = 3 weeks after day 1, day 3 = 4.5 months after day 1. The tubes were centrifuged for 5 minutes at 2000 g and the plasma was removed. To remove any extracellular NSE, the remaining cells were washed three times by adding an equal volume of physiological salt. The cells were then dissolved in physiological saline and lysed by freezing them for at least 2 hours at –80°C. After thawing and centrifugation for 5 min at 2000 g, the cellular debris was removed and dilutions of 0%, 1%, 2%, 2.5%, 3%, 4%, 5%, 6%, 10%, 20%, 30% and 40% hemolysate in physiological saline were prepared.

### Quantification of NSE and hemolysis

NSE was quantified with the use of a commercially available electrochemiluminescent assay (ECLIA) on two different Cobas platforms (e602 and e801, Roche Diagnostics, Rotkreuz, Switzerland). In this assay, both the γγ homodimer and the αγ heterodimer are detected simultaneously. The supplier reported expected values within a healthy population of 15.7–17.0 ng/mL (95% confidence range). In case of the Cobas e602, the supplier reported within-lab CV values of 2.2%, 3.1% and 3.8% measured with the use of pooled human serum samples with mean NSE concentrations of 0.87, 11.4 and 87.3 ng/mL respectively (*n* = 60). Within-lab CV values of 6.7%, 1.3% and 1.6% were reported when human serum samples with mean NSE concentrations of 0.411, 20.4 and 194 ng/mL were measured on the Cobas e801. The test was validated on both platforms and provided comparable results (data not shown).

The amount of hemolysis was expressed using the H-index, a measure that corresponds with the Hb concentration (1H-index = 1 mg/dL = 0.621 μmol/L Hb). The H-index was determined *via* an absorbance assay (wavelengths 570 and 600 nm) on two Cobas platforms (Serum index Gen.2, Cobas c702 and Cobas c501, Roche Diagnostics). The manufacturer reported CV values of 27.0%, 0.9% and 0.7% for the Cobas c702 platform, determined in human serum samples with mean H-index values of 4.71, 127 and 362 μmol/L respectively (*n* = 21). CV values of 16.8%, 1.2% and 0.3% were reported when human serum samples with mean H-index values of 3.93, 155 and 307 μmol/L were measured on the Cobas c501 platform. The test was used in daily clinical practice and validated on both platforms, which provided comparable results (data not shown).

### Derivation of a hemolysis correction equation

Serum pool 1 and 2 with initial NSE concentrations of 33.3 and 18.1 ng/mL were prepared by combining residual serum samples with low H-indices (final H-indices of 0 and 1.9 μmol/L respectively). Each serum pool was divided into several aliquots and spiked with the following hemolysate dilutions in a 1:10 ratio: 0%, 1%, 2%, 3%, 4%, 5% and 6% in case of serum pool 1 and 0%, 2.5%, 5% and 10% in case of serum pool 2. The NSE concentrations and corresponding H-indices were measured on the Cobas e801 and c501 platform and analyzed using least-squares linear regression. The results of the individual pools were combined by transforming the data of the individual pools through the origin (0,0) with the use of ΔNSE (NSE_measured_ – intercept), after which it was plotted as a function of H-index and analyzed *via* least-squares linear regression.

### Validation of the hemolysis correction equation

A training and validation dataset were used to evaluate the performance of the obtained correction equation. The training dataset was based on the results obtained with the five hemolysate pools used to construct the correction equation, while the validation dataset was made by spiking serum pool 1 and 2 in a similar fashion with five new hemolysate pools from other individuals (pool f–j). The H-indices and NSE concentrations of all validation samples were measured on the Cobas e801 and c501 platform. To verify the applicability of the correction equation on both Cobas platforms, the samples of pool f were measured on the Cobas e602 and c702 platform as well (Supplementary Figure 1A). Corrected NSE values were derived by applying the hemolysis correction equation to the results of the intentionally hemolyzed serum samples of the training and validation datasets. The TEa was calculated using the following formula: 1.65·0.50·CV_i_+0.25·(CV_i_^2^+CV_G_^2^)^0.5^, with CV_i_ = 10.9% and CV_G_ = 20.3%, both previously determined using similar detection platforms as the ones used in this research [27].

### Study design and participants

This study protocol was approved by the Medical Research Ethics Committees United, the Netherlands. During a multicenter prospective study, patients suspected of lung carcinoma were included by their primary care physician in one of the following hospitals: Catharina Hospital Eindhoven (*n* = 176), Máxima Medical Center Eindhoven/Veldhoven (*n* = 80), Amphia Hospital Breda (*n* = 42), Sint Anna Hospital Geldrop (*n* = 10) and Sint Jans Gasthuis Weert (*n* = 8). After written informed consent, serum was collected prior to the final diagnosis and start of any therapeutic action. Yet, some patients received treatment for other common chronic diseases such as chronic obstructive pulmonary disease, asthma, cardiovascular diseases, diabetes mellitus etc. The diagnosis of lung cancer was established by using standard clinical workup procedures and lung cancer subtypes were classified *via* cytological and/or histological examination according to World Health Organization criteria [28]. Patients with other primary tumors were excluded. The full study protocol can be requested from the corresponding author.

### Applying the hemolysis correction equation in SCLC diagnostics

The serum NSE concentrations and H-indices of all patients included in the study (*n* = 316) were determined using the Cobas e602 and c702 platform. The influence of the hemolysis correction equation was assessed by applying the correction equation to all samples except for those having an H-index above 30 μmol/L (*n* = 2).

### Statistical analysis

Python 3.7 was used to evaluate the obtained results and perform statistical analyses. Normality was evaluated by QQ-plots and the Shapiro-Wilk test. Since both tests did not confirm normality, a non-parametric Wilcoxon signed rank sum test was used to compare the differences between the baseline, uncorrected and corrected samples. A paired *t-test* was used to evaluate the differences between the baseline samples and the subgroups of the corrected samples, since normality was confirmed. A Mann-Whitney-U test was performed to evaluate the differences between the uncorrected NSE values of the different patient groups and differences between measured and corrected NSE levels were analyzed with a non-parametric Wilcoxon signed rank sum test due to non-normality. Receiver operating characteristic (ROC) curves and the area under the curve (AUC) were constructed *via* stratified bootstrapping (resampling with replacement 100 times). AUCs were shown as mean ± 2SD. A paired *t-test* was used to evaluate the difference between the AUCs. For each bootstrap iteration the Youden’s J statistic (Youden’s J = sensitivity + specificity -1) was determined to derive optimal cut-off values, which were combined to one optimal cut-off value (median + IQR). Since the data was not assumed to be normally distributed, a Wilcoxon signed rank sum test was used to evaluate the difference between the cut-off values of uncorrected and corrected NSE. Sensitivity, specificity, positive predictive value (PPV) and negative predictive value (NPV) of each bootstrap were calculated using standard formulas and the median and IQR of all bootstraps was shown. A *p-value* less than 0.05 was considered statistically significant.

## SUPPLEMENTARY MATERIALS



## Abbreviations

NSE: neuron-specific enolase
SCLC: small-cell lung cancer
NSCLC: non-small cell lung cancer
ProGRP: progastrin-releasing peptide
Hb: hemoglobin
H-index: hemolysis index
SD: standard deviation
TEa: total allowable analytical error
CV_a_: analytical variation
ECLIA: electrochemiluminescent assay
ROC: receiver operating characteristic
AUC: area under the curve
IQR: inter-quartile range
CV_G_: between-subject variation
CV_i_: within-subject biological variation
PPV: positive predictive value
NPV: negative predictive value
