# Supplementary material for: Correction of the NSE concentration in hemolyzed serum samples improves its diagnostic accuracy in small-cell lung cancer
Source: Oncotarget. 2020 Jul 7;11(27):2660–8. doi: 10.18632/oncotarget.27664 (PMC7343637; doi:10.18632/oncotarget.27664)
Supplement: Supplementary file 1 [file oncotarget-11-2660-s001.pdf]

Correction of the NSE concentration in hemolyzed serum samples improves its diagnostic accuracy in small-cell lung cancer

SUPPLEMENTARY MATERIALS

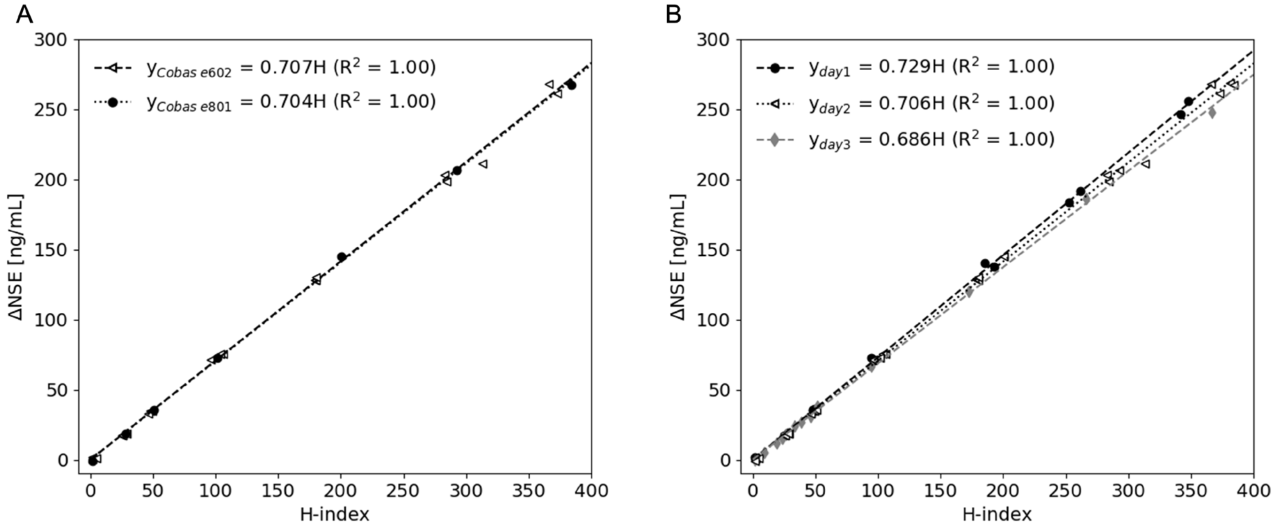

Supplementary Figure 1: Analytical and intra-individual validation. The ΔNSE concentration and corresponding H-index of the samples of hemolysate pool f (**A**) measured on both the Cobas e602/c702 and Cobas e801/c501 platforms and (**B**) measured at three different time points (day 1 = time point 0, day 2 = 3 weeks after day 1, day 3 = 4.5 months after day 1), indicating identical slopes within range of the CV_a.

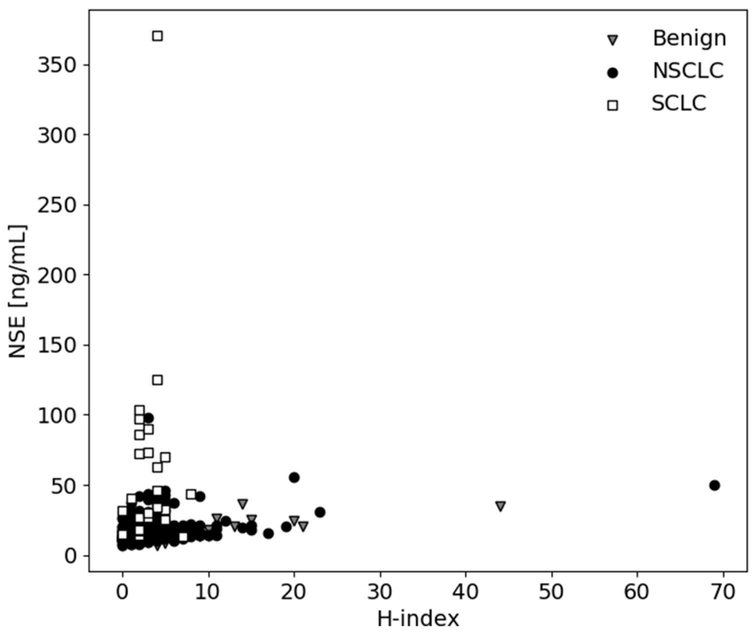

Supplementary Figure 2: The measured NSE concentration and corresponding H-indices of all patients included in the study, divided into the groups benign: triangle, NSCLC: circle, SCLC: square.

Supplementary Table 1: Derived equations with corresponding SD of the slope, intercept, R^2 and the SD of the residuals of each individual pool (1a–e and 2a–e) and the hemolysis correction equation derived after combining all pools

Pool	Equation, ΔNSE =	R^2	SD of residuals
1a	32.94 (± 0.479) + 0.444 (± 0.016) H-index	0.994	0.600
1b	33.23 (± 0.480) + 0.463 (± 0.016) H-index	0.994	0.585
1c	33.44 (± 0.472) + 0.624 (± 0.016) H-index	0.997	0.585
1d	33.33 (± 0.114) + 0.300 (± 0.003) H-index	0.999	0.139
1e	33.00 (± 0.434) + 0.551 (± 0.014) H-index	0.997	0.526
2a	16.99 (± 0.334) + 0.440 (± 0.007) H-index	1.000	0.300
2b	16.95 (± 0.246) + 0.469 (± 0.005) H-index	1.000	0.222
2c	16.56 (± 0.321) + 0.632 (± 0.006) H-index	1.000	0.286
2d	17.56 (± 0.102) + 0.294 (± 0.002) H-index	1.000	0.091
2e	16.76 (± 0.271) + 0.552 (± 0.005) H-index	1.000	0.242
Combined	0.469 (±0.016) H-index	0.864	4.796

Supplementary Table 2: Overview of the diagnosis, characteristics and laboratory results of all patients included in the analysis

Characteristics	Benign	SCLC	NSCLC
**Total number of patients**	75	26	215
**Females (%)**	40.0	50.0	49.8
**Age (years)**	66 (60–72)	70 (63–71)	69 (62–73)
**NSE measured (ng/mL)**	14.0 (11.7–15.5)	37.4 (25.6–73.2)	14.7 (12.6–19.6)
**H index measured (µM)**	4 (2–6)	3 (1.5–5)	3 (2–4)
**NSE corrected (ng/mL)**	11.6 (9.8–13.7)<br>*n* = 74	36.1 (23.5–71.9)<br>*n* = 26	12.9 (10.7–17.6)<br>*n* = 214

Alternative diagnoses	
COPD	8
Pneumonia	12
Tuberculosis	1
Heart disease	1
Bronchiectasis	1
Other benign diseases	3
Other carcinomas	7
No other diagnosis	16

Data is presented as *n* (exact number) or median (IQR) unless otherwise stated. Significant differences between benign, SCLC and NSCLC were observed in case of NSE concentration and the difference between measured and corrected NSE.

Supplementary Table 3: Sensitivity, specificity, PPV and NPV obtained when using uncorrected and corrected NSE in SCLC diagnostics with both the conventional and newly determined cut-off value

	Cut-off 24.5 ng/mL		Cut-off 22.7 ng/mL	
	**Corrected**	**Uncorrected**	**Corrected**	**Uncorrected**
**Sensitivity (%)**	73.1 (69.2–76.9)	76.9 (73.1–80.8)	76.9 (73.1–80.8)	76.9 (73.1–80.8)
**Specificity (%)**	90.3 (88.9–91.3)	88.5 (87.2–89.6)	88.9 (87.2–90.3)	85.8 (84.9–87.5)
**PPV (%)**	40.0 (37.0–43.5)	37.7 (35.1–41.2)	38.2 (35.7–42.0)	32.8 (31.1–36.6)
**NPV (%)**	97.4 (97.0–97.8)	97.7 (97.3–98.1)	97.7 (97.3–98.1)	97.6 (97.2–98.1)

Data is presented as median (IQR).
